# A Randomized, Controlled Trial Comparing the Impact of a Low-Calorie Ketogenic vs a Standard Low-Calorie Diet on Fat-Free Mass in Patients Receiving an Elipse™ Intragastric Balloon Treatment

**DOI:** 10.1007/s11695-020-05133-8

**Published:** 2020-11-20

**Authors:** Luigi Schiavo, Giovanni De Stefano, Francesco Persico, Stefano Gargiulo, Federica Di Spirito, Giulia Griguolo, Niccolò Petrucciani, Eric Fontas, Antonio Iannelli, Vincenzo Pilone

**Affiliations:** 1 Department of Medicine, Surgery, and Dentistry, “Scuola Medica Salernitana”, University of Salerno, Fisciano, SA Italy; 2 Center of Excellence of Bariatric Surgery of the Italian Society of Obesity Surgery and Metabolic Disease (SICOB), Unit of General and Emergency Surgery, University Hospital San Giovanni di Dio e Ruggi d’Aragona, Mercato San Severino, Salerno, Italy; 3 Med.Ita Advanced Biomedical Solutions, Naples, Italy; 4 Unit of General and Specialistic Surgery, A.O.R.N. dei Colli Ospedali Monaldi-Cotugno-CTO, Naples, Italy; 5 General Surgery Unit, Santa Maria La Bruna Clinic, Torre del Greco, Italy; 6 Digestive Unit, Archet 2 Hospital, University Hospital of Nice, F-06202 Nice, France; 7 Direction de la Recherche Clinique, University Hospital, Nice, France; 8 Inserm, U1065, Team 8 “Hepatic Complications of Obesity”, F-06204 Nice, France; 9 University of Nice Sophia-Antipolis, F-06107 Nice, France

**Keywords:** Obesity, Elipse™ intragastric balloon, Fat-free mass, Resting metabolic rate, Low-calorie ketogenic diet

## Abstract

**Background:**

The Elipse™ intragastric balloon (EIGB) is a new swallowable balloon for weight loss (WL). Preserving metabolically active fat-free mass (FFM) and resting metabolic rate (RMR) during WL are crucial to maximize fat mass (FM) loss. After EIGB placement, a standard low-calorie diet (LCD) is generally prescribed. A low-calorie ketogenic diet (LCKD) has proven to be safe and effective in reducing FM while preserving FFM and RMR.

**Objective:**

To prospectively compare the effects on WL, FM, FFM, and RMR in two groups of patients who were randomized to two different diets: LCKD and a standard LCD after EIGB placement.

**Methods:**

WL, FM, FFM, and RMR were measured before EIGB and at 4 months in 48 patients who received either a LCKD (*n* = 24) or a standard LCD (*n* = 24). Compliance in following the prescribed diet was determined with food frequency questionnaires in all patients. The impact of LCKD and LCD on renal function was also evaluated.

**Results:**

The LCKD group showed a significantly lower decrease in FFM and RMR when compared with the LCD group (3.55 vs 14.3%, *p* < 0.001; 9.79 vs 11.4%, *p* < 0.001, respectively). FM decreased more significantly with LCKD compared to LCD (41.6 vs 33.1%, *p* = 0.0606). Compliance in following the prescribed diets, without negative impact on renal function, was found.

**Conclusion:**

Based on our findings, despite the small sample size, we were able to support the hypothesis that LCKD is associated with an increased FM loss while reducing the FFM loss and the RMR, without interfering with renal function after EIGB.

## Introduction

Intragastric balloons have been used for weight loss (WL) in the last two decades, but the need for hospital recovery, upper GI endoscopy, and anesthesia is major limitations for this technique. The Elipse™ intragastric balloon (EIGB) is a new swallowable balloon that has no such limitations and has been proven to be safe, effective in achieving weight loss is also, and well tolerated by patients [1–6].

After EIGB placement, a standard low-calorie diet (LCD) program, based on a daily intake of about 1000–1200 kcal/day, is generally prescribed [6]. The combination of the restrictive effect of EIGB and diet accounts for a significant loss of fatty free mass (FFM). Indeed, maintaining adequate FFM is an important consideration when making WL because muscles play a central role in whole-body protein metabolism [7]. Additionally, a significant decrease in FFM may negatively affect the resting metabolic rate (RMR) [8–11], slow the rate of WL, and predispose to weight regain [12]. Moreover, no studies have been conducted so far on the amount of WL attributed to the loss of fat mass (FM) and FFM in the setting of EIGB treatment.

A low-calorie ketogenic diet (LCKD) has been proven to be safe and effective for WL [13–17], especially in reducing FM while preserving FFM and RMR [18–20].

Therefore, we designed a randomized controlled trial to compare the effect of LCKD and a standard LCD after EIGB on WL, FM, FFM, and RMR.

## Materials and Methods

### The Elipse™ Intragastric Balloon System

The EIGB (Allurion Technologies, Natick, MA, USA) is a swallowed, self-emptying, and excreted gastric balloon for WL [21]. It is folded into a vegetarian capsule and attached to a thin catheter. The capsule is easily swollen with a glass of water. However, in case swallowing is problematic, a stylet can be fed through the catheter to stiffen it, allowing the physician to gently push the capsule during swallowing. The EIGB contains a small radiopaque ring that can be used to confirm its correct position inside the stomach through an abdominal X-ray [21]. Once the capsule is in the stomach, the balloon is filled with 550 mL of liquid consisting distilled water with potassium sorbate preservative, and then the catheter is removed, and a second X-ray is performed to confirm the balloon is filled and that the placement is complete [21]. After approximately 4 months, a time-activated release valve opens, allowing the balloon to empty and pass naturally through the digestive system to be excreted and thus ideally does not require any endoscopic procedure [22].

### Pre- and Post-EIGB Placement Medical Treatment

Patients fasted for at least 8 h prior to the EIGB placement and were given proton pump inhibitors, anti-emetics, and anti-nausea and vomiting drugs before and after the EIGB placement. Details are reported in Table 1.

**Table 1:** Medical Therapy before and after the EIGB placement

Medicaments	Timing
Pantoprazole (Pantorc®) (40 mg) (Administered per os)	7 days prior the EIGB placement and through the end of the balloon treatment (4 months)
Aprepitant (Emend®) (125 mg) (Administered per os)	The evening before the EIGB placement and then for 3 consecutive days after the balloon placement
Ondansetron (Zofran®) (8 mg) (Administered per os)	2 h prior the EIGB placement; 6 h after the balloon placement, and then for 3 consecutive days after the EIGB treatment

### Study Design and Patient Selection

Between 2018 and 2019, we conducted a pilot, prospective, randomized, controlled trial on a cohort of consecutive obese individuals undergoing EIGB at our institutions. In accordance with other studies, the EIGB inclusion criteria were as follows: body mass index (BMI) ≥ 27 kg/m^2^, and less than 45 kg/m^2^, and age between 18 and 65 years. Exclusion criteria included previous bariatric or gastric surgery, bowel obstruction, hiatal hernia >5 cm, heart failure, blood coagulation disorders, more than one other abdominal/gynecological operation, certified pregnancy, eating disorders (bulimia, binge eating disorder, or night eating syndrome) [1–6], serum creatinine level greater than 1.8 mg/dL or liver enzyme (glutamic oxaloacetic transaminase (GOT) or glutamic pyruvic transaminase (GPT)) levels less than three times the upper limit of normal [17], inability to comply with the LCKD or LCD for religious reasons, or the presence of chewing or swallowing disorders. After EIGB placement, the patients were randomized into two groups: the LCKD group that followed a LCKD and the LCD group that followed a standard LCD. Considering the small sample size, to achieve balance in the allocation of patients to treatment arms and to increase the probability that each arm contained an equal number of patients, block randomization was used [23]. Informed written consent was obtained from each participant after being informed about the purpose and nature of the study (Research Registry Unique Identifying Number 5478).

### Primary and Secondary Outcomes

The primary outcome was the amount of FFM loss. Secondary outcomes included body weight (BW), FM, RMR, and dietary compliance*.*

### LCKD and LCD Characteristics

Patients were discharged 2–4 h after the EIGB placement. With regard to the dietary recommendations, only fluid hydration was permitted for the first 24 h. During the first week, a gradual progression to a semi-liquid diet (yogurt, mashed potatoes, thin soup, and puréed vegetables) was recommended in both groups. At the beginning of the second-week post-EIGB placement, the patient proceeded with caution to a hypocaloric, textured diet plan. Regular and moderate physical activity was suggested to all patients (30 min day^−1^). At this time, patients enrolled for the study were randomized in two groups: a LCKD group (experimental group) and a standard LCD group (control group). Both regimens were applied until the end of the treatment after a detailed discussion on both diet schemes with the nutritionist. To ensure that all included patients consumed a similar diet, we developed two meal plans (LCKD and LCD), assigning a specific quantity to individual foods using a free online keto diet application (https://www.eatthismuch.com) and the Nutrigeo 8 software (Progeo, Ascoli Piceno, Italy) for the LCKD plan and the LCD plan, respectively.

**Fig. 1:**
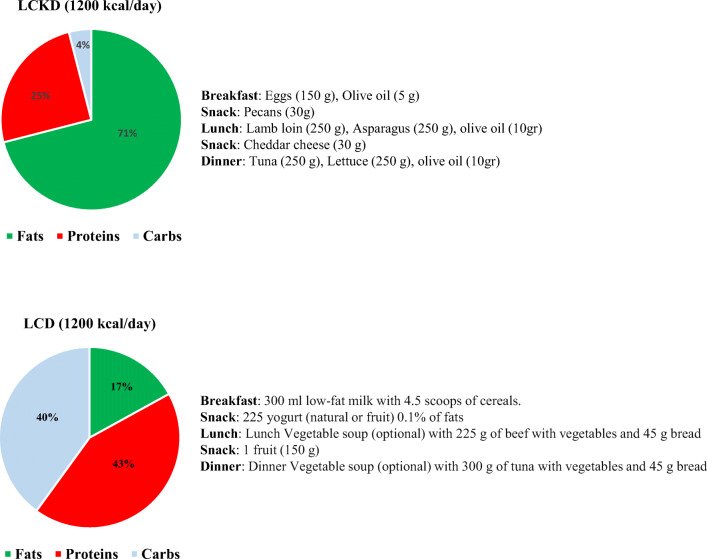
Sample 1200 kcal intervention daily menu for low-calorie ketogenic diet (LCKD) and low-calorie diet (LCD) group

The macronutrients composition of the LCD and LCKD was 40% carbohydrates, 43% proteins, and 15% fats (~ 1200 kcal/day) [24], and 4% carbohydrates, 25% proteins, and 71% fats (~ 1200 kcal/day) [17], respectively. An example of the LCKD and LCD daily plan is reported in Fig. 1.

### BW, FM, FFM, RMR, and Laboratory Determinations

**Fig. 2:**
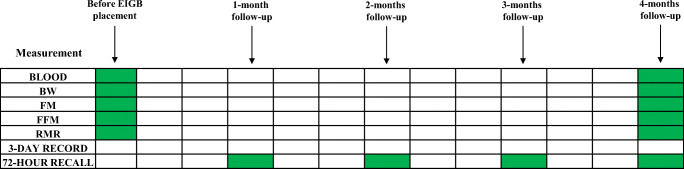
Time periods of blood sampling, body weight (BW), fat mass (FM), fat-free mass (FFM), resting metabolic rate (RMR), 3-day record, and 72-h recall during the Elipse™ intragastric balloon (EIGB) treatment

The details regarding the sequence of measurements are shown in Fig. 2. BW (kg) and height (cm) were determined under standard conditions. Height was measured using a Seca 206 mechanical measuring tape (Intermed, Milano, Italy); BW were assesed by the Seca 869 flat digital scale (capacity 250 Kg, Intermed, Milano, Italy). Patients’ body composition was measured by bioelectrical impedance assay (BIA) using the Jawon IOI 353 Segmental Body Composition Monitor (Cosmed, Italy) [9, 11, 17]. The instrument used is the last generation in body composition analysis, using the latest multi-frequency technology, and it is in compliance with the requirements of the Directive 90/384/EEC for weighing with non-automatic devices in the medical sector and the Directive 93/42/EEC for medical devices. To perform an appropriate analysis, as we previously reported [8, 9, 11, 16, 25], all patients were required to comply with these conditions prior to the BIA: no food ingestion for at least 4 h, minimal intake of 2 L of water the day before, no physical activity for at least 8 h, no coffee or alcoholic beverage consumption during at least 12 h, and no diuretic use for at least 24 h. Patients were also asked to empty their bladder immediately prior to the BIA test. Patient’s RMR were measured by indirect calorimetry using Fitmat PRO monitor (Cosmed, Italy) [11, 17]. Examinations were performed from 8:00 to 10:00 a.m. in the same room under thermos neutral conditions, in order to reduce diurnal variation between subjects [26–29]. Measurements were performed at a duration of 15 min following a prior 5–10-min test.

Blood tests included liver enzymes (GOT, GPT, and gamma**-**glutamyl transferase (GGT)), glucose, insulin, creatinine, urea, and glomerular filtration rate (GFR), uric acid, urea nitrogen, ketonemia, iron, hemoglobin, total cholesterol, high-density lipoprotein (HDL), low-density lipoprotein (LDL), and triglycerides. The GFR was calculated using the Modification of Diet in Renal Disease formula [30]. All blood analyses were performed in an approved laboratory with internal and external quality controls using the reagents provided by the manufacturer and following the manufacturer’s instructions. Data were compared with accepted clinical cutoff values (Table 2).

**Table 2:** Patients’ clinical parameters before EIGB placement and after 4 months follow-up

Clinical parameters	Diet	Pre-EIGB placement	Post-EIGB (follow-up 4 months)	*p* value
Triglycerides (mg/dL) [≤ 230]	LCKD	220 ± 36	114 ± 15.2	< 0.001*
	LCD	202 ± 21.2	93 ± 8.3	< 0.001*
Total cholesterol (mg/dL) [< 200]	LCKD	226 ± 15.6	164 ± 43.4	< 0.001*
	LCD	210 ± 27.4	135 ± 27.5	< 0.001*
HDL (mg/dL) [30–70]	LCKD	38.5 ± 7.6	71 ± 8.3	< 0.001*
	LCD	33.8 ± 8.3	83 ± 6.5	< 0.001*
LDL (mg/dL) [< 150]	LCKD	147 ± 25.3	78.2 ± 11.4	< 0.001*
	LCD	138 ± 26.2	41.4 ± 17.9	< 0.001*
Glucose (mg/dL) [65–110]	LCKD	123 ± 10.2	82 ± 18.9	< 0.001*
	LCD	130 ± 24.7	92 ± 26.9	< 0.001*
Insulin (mU/L) [< 25]	LCKD	11.4 ± 7.9	4.3 ± 1.9	< 0.001*
	LCD	8.9 ± 5.4	5.3 ± 2.3	< 0.001*
GOT (U/L) [10–59]	LCKD	50 ± 8.1	24 ± 10.4	< 0.001*
	LCD	40 ± 9.9	26 ± 7.4	< 0.001*
GPT (U/L) [0–50]	LCKD	41 ± 10.3	28 ± 11.2	< 0.001*
	LCD	37 ± 8.3	22 ± 8.9	< 0.001*
GGT (U/L) [10–50]	LCKD	28 ± 16.2	14 ± 3.4	< 0.001*
	LCD	29 ± 9.8	22 ± 6.7	< 0.001*
Creatinine (mg/dL) [0.5–1.3]	LCKD	1.15 ± 0.5	0.92 ± 1.2	0.391**
	LCD	0.94 ± 0.3	1.12 ± 1.4	0.541**
Uric acid (mg/dL) [2.4–7]	LCKD	5.4 ± 2.5	4.60 ± 3.2	0.339**
	LCD	5.9 ± 2.9	6.3 ± 2.4	0.605**
Urea nitrogen (mg/dL) [5 to 20]	LCKD	19 ± 8.2	16 ± 8.4	0.217**
	LCD	17 ± 7.2	19 ± 7.6	0.354**
Ketonemia (mmol/L) [0–0.6]	LCKD	0.02 ± 0.03	0.18 ± 0.2	< 0.001*
	LCD	0.04 ± 0.02	0.047 ± 0.05	0.527**
Iron (ng/dL) [65–175]	LCKD	96 ± 16.2	93 ± 13.4	< 0.488**
	LCD	85 ± 11.4	79 ± 11.7	< 0.0785**
Hb (g/dL) [12.5–18]	LCKD	13.4 ± 4.5	12.7 ± 5.4	0.627**
	LCD	14.6 ± 6.9	12.5 ± 7.8	0.328**

EIGB, Elipse intragastric balloon

LCKD, low-calorie ketogenic diet

LCD, low-calorie diet

HDL, high-density lipoprotein

LDL, low-density lipoprotein

GOT, glutamic oxaloacetic transaminase

GPT, glutamic pyruvic transaminase

GGT, gamma-glutamyl transferase

Hb, hemoglobin

*p < 0.05); **p ≥ 0.05

### Dietary Compliance Assessment Methods

Nutritional assessment and dietary counseling were scheduled at 1, 2, 3, and 4 months after EIGB placement (Fig. 2). Dietary assessments were primarily performed using questionnaires (3-day estimated food records and 72-h recalls) [31–33]. Nutrient intakes were calculated from the 72-h recalls and 3-day dietary records (Sunday to Tuesday; breakfast to bedtime) using the Nutrigeo 8 software.

### Statistics

#### Sample Size Calculation and Statistical Analysis

Based on our previous investigations (personal unpublished data) and literature review, we made the hypothesis that patients in the LCD would lose 10 (± 3) kg of FFM and those in the experimental group (LCKD), only 7 kg. Using a priori power analysis (G*Power Software, Dusseldorf, Germany), we found that a sample of 21 patients in each arm could detect a difference in the primary outcome between the groups, with 90% power and an alpha error of 5%. We planned to include 24 patients per group considering 15% drop out.

The effects of post-EIGB placement LCKD and LCD diet program were directly compared by using the paired sample *t*-test for continuous variables for comparison within groups and the Mann–Whitney test for comparison between LCKD and LCD groups. The pattern of BW, FM, FFM, and RMR changes during the period study was expressed as a percentage and plotted over time. Nutrient intake comparison between the prescribed diets, the 3-day estimated food records, and the 72-h recalls were performed by analysis of variance (ANOVA). Block randomization was performed using a free online Graph Pad Quick Calcs Software (https://www.graphpad.com/quickcalcs/randomize1/). Data are reported as mean ± standard deviation (SD). A *p* value < 0.05 was considered statistically significant. Furthermore, any *p* value less than 0.001 was conventionally stated merely as *p* < 0.001. IBM SPSS Statistics for Windows (Version 25.0. Armonk, NY: IBM Corp) was used for statistics.

## Results

### Characteristics of the Study Population

**Fig. 3:**
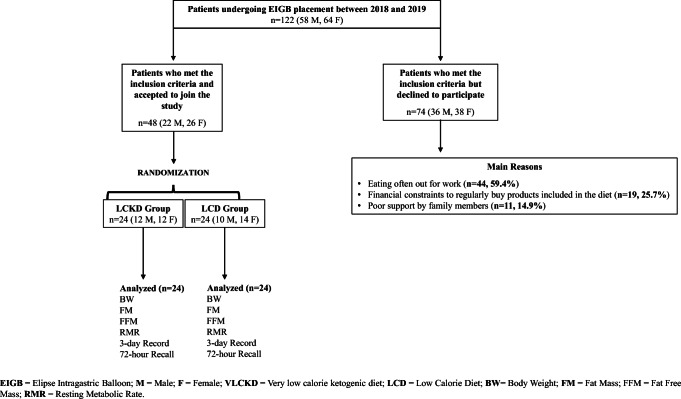
Flow chart of patient’s selection

Figure 3 reports the flow chart of patient’s selection. Of 122 consecutive patients (64 females, 58 males), 48 (26 females, 22 males) met the inclusion criteria and accepted to join the study whereas 74 (36 M, 38 F) did not (Fig. 3).

Pre-EIGB placement mean age and BMI were 39 ± 8.8 years and 37.8 ± 4.9 kg/m^2^ in the LCKD group, and 41 ± 4.3 years and 37.2 ± 6.4 kg/m^2^ for the LCD group, respectively. Before EIGB placement, LCKD and LCD groups were comparable in terms of BW, FM, and FFM (*p* = 0.227, 0.496, and 0.463, respectively) whereas RMR was significantly higher in the LCKD group (*p* = 0.0232) (Table 3). No patients dropped out the study. As expected, we observed that BW, FM, FFM, and RMR significantly decreased after 4 months follow-up in both groups (LCKD (*p* < 0.001, *p* < 0.001, *p* = 0.0260, and *p* < 0.001, respectively) and LCD (*p* < 0.001, *p* < 0.001, *p* = 0.0232, and *p* < 0.001, respectively)) (Table 3).

**Table 3:** Body weight (BW), fat mass (FM), fat-free mass (FFM), resting metabolic rate (RMR), and phase angle at baseline and after EIGB (4 months). Data are reported as median (range)

Parameters	Diet	Pre-EIGB	Post-EIGB (follow-up 4 months)
TBW (kg)	LCKD	112 (90–122) ^a^	91.8 (72.8–100) ^c^
	LCD	106.5 (98–125)	84.1 (77.4–98.7) ^c^
FM (kg)	LCKD	37.5 (15–51.2) ^a^	21.9 (6.9–34) ^c^
	LCD	36.2 (28–44)	24.2 (17–38.1) ^c^
FFM (kg)	LCKD	70.5 (65–78) ^a^	68 (55–75) ^c^
	LCD	70 (64.7–82.5)	60 (48–66) ^c^
RMR (kcal/day)	LCKD	2170 (1885–2456) ^b^	1957.5 (1827–2175) ^c^
	LCD	2030 (1875–2392)	1798 (1440–1972) ^c^
Phase Angle (°)	LCKD	7.1 (5.7–7.7) ^a^	6.0 (4.7–6.5) ^c^
	LCD	7.0 (6.2–8)	5.5 (5.1–6.4) ^c^

LCKD, low-calorie ketogenic diet

LCD, low-calorie diet

EIGB, Elipse™ intragastric balloon

^a^*p* ≥ 0.05 vs pre-EIGB LCD

^b^*p* < 0.05 vs pre-EIGB LCD

^c^*p* < 0.05 vs pre-EIGB

### Performance, Safety, and Side Effects

93.7% of patients (45/48) swallowed the Elipse™ capsule with a glass of water, whereas three patients (6.3%) required assistance with a stylet. There were no complications during capsule passage. All EIGBs were visualized successfully on X-ray before and after filling. All patients were successfully discharged 2–4 h after the EIGB placement. The most common adverse events after EIGB placement were nausea and vomiting (73% (35/48) and 50% (24/48) of patients, respectively). All nausea and vomiting were either self-limiting or resolved with medications (Table 1) in 2–3 days. At the end of the treatment, all EIGBs were naturally excreted in the stool.

### Impact of LCKD and LCD on FFM and RMR

As shown in Table 3, patients that followed the LCKD lost less FFM and RMR at 4 months after EIGB placement than patients who followed the LCD (3.55 vs 14.3%, *p* < 0.001; 9.79 vs 11.4%, *p* < 0.001, respectively).

### Impact of LCKD and LCD on BW and FM

Patients in the LCKD group lost significantly more FM at 4 months after EIGB placement than patients who followed the LCD (41.6 vs 33.1%, *p* = 0.0606, despite a significantly lower WL (18 vs 21%, *p* < 0.001, Table 3).

### Impact of LCKD and LCD on patient’s Clinical Parameters

As shown in Table 2, both LCKD and LCD patients showed a clear improvement in patients’ clinical status, including liver enzyme levels (GOT, GPT, and GGT), glycemic profile (glucose and insulin), and lipid profile (total cholesterol, HDL, and LDL, and triglycerides), without detecting any significant deviation in biochemical kidney parameters (creatinine, urea, and GFR) and uric acid levels. Furthermore, after the 4-month LCKD, patients had significantly higher blood ketone levels compared to baseline (*p* < 0.001) whereas in LCD group only a not significant trace of blood ketones was found compared to baseline (*p* = 0.527) (Table 2).

### Three-Day Estimated Food Records vs 72-h Recalls

No significant differences in the estimated nutrient intake were observed between the post-EIGB placement diet prescription, the 72-h recalls, and the 3-day estimated food records in both LCKD and LCD groups. Values for energy intake (expressed in kcal/day) and all macronutrients reported during the 72-h recalls and the three-day estimated records were strictly similar to those of the post-EIGB placement, indicating a high patient’s compliance of following the prescribed diets in both group studies (Table 4).

**Table 4:** Macronutrients daily intake from the 3-day estimated food record and the 72-h recall of the participants consuming a low-calorie ketogenic diet (LCKD) or a low-calorie diet (LCD). *p* < 0.05

Parameter	LCKD	3-day estimated food record	72-h recall	*p*
	Prescribed food plan	Follow-up, 4 months	Follow-up, 4 months	
Energy (kcal)	1200	1270 ± 41.3	1250 ± 30.9	0.0637
Protein (%)	25	28 ± 5.3	26.2 ± 7.2	0.329
Carbohydrate (%)	4	4.8 ± 0.87	4.6 ± 2.4	0.703
Fat (%)	71	67.2 ± 15.2	69.2 ± 21.8	0.714
Energy (kcal)	1200	1235 ± 31.9	1255 ± 46.3	0.0881
Protein (%)	43	48 ± 10.4	46.2 ± 17.2	0.663
Carbohydrate (%)	40	43.7 ± 20.3	45.4 ± 22.9	0.787
Fat (%)	15	8.3 ± 3.5	8.4 ± 2.8	0.913

## Discussion

Based on our findings, despite the small sample size, this study indicates that LCKD is associated with an increased FM loss while reducing the FFM loss and the RMR, without interfering with renal function after EIGB. These findings are in accordance with other studies who confirmed that the ketogenic diet is safe and highly effective in terms of BW reduction without inducing a significant FFM loss [18, 34].

Interestingly, herein, we found that patients who followed the LCD had a greater total body weight loss, at 4 months after EIGB, than patients who followed the LCKD. Our findings indicate that the higher weight loss in the LCD group was mainly due to FFM loss and less FM than the experimental group. The net result of the body composition changes is a greater total body weight decrease in the LCD group. Our results indicate that the process of body weight loss is more physiologic in the experimental group that loses less FFM than the control group. Among the bioimpedance parameters measured with BIA, the phase angle (defined as the ratio of resistance (intracellular and extracellular resistance) to reactance (cell membrane-specific resistance) expressed as an angle) is a clinically important parameter used for nutritional assessment and for assessment of the risk of various disease [35]. Interestingly, as shown in Table 3, herein, we found that patients who followed the LCD had a lower phase angle, at 4 months after EIGB placement, than patients who followed the LCKD. In our opinion, this finding is clinically relevant considering that the phase angle represents both the amount and quality of soft tissue, with a high phase angle reflecting higher cellularity, better cell heath and better nutritional status [35].

Furthermore, the sustained weight and FM loss induced by LCKD did not induce any significant reduction in RMR, probably due to the preservation of FFM. In particular, we show that in the LCKD group, the RMR was preserved and remained within the expected limits for the variation in FFM. Interestingly, the metabolic adaptation phenomenon called “adaptative thermogenesis,” defined as a decrease in RMR out of proportion to the decrease in body mass was not activated in concomitance with the LCKD [20, 36, 37]. On the contrary, patients in the LCD group in addition to the significant weight and FM loss showed a significant decrease in both FFM and RMR.

RMR is recognized as the major component of total energy expenditure, being responsible for about 75% of daily total energy expenditure [38]. Therefore, any RMR reduction-induced WL translates into a large impact on energy balance, making subjects more prone to weight regain over time [12, 20]. In agreement with Gomez-Arbelaez et al., herein, we found that the most plausible reason accounting for the not significant reduction of RMR seen in patients who had the LCKD after the EIGB is the preservation of FFM [20]. Preservation of initial RMR after WL could play a key role in preventing weight regain in the short and long time [39].

In the present study, we found that before EIGB placement, RMR was slightly but significantly higher in the LCKD group than that in the LCD group. However, this data is expected because despite the fact that it is widely accepted that FFM is the major factor determining RMR [40], other factors, such as hormonal status and age, influence the RMR [40].

LCKD appears to be protective against muscle mass catabolism for at least three reasons: first, low blood sugar’s level inhibits the muscle proteolysis; secondary, ketone bodies suppress the use of protein-derived amino acid by muscle; third, the β-hydroxybutyrate (the main ketone body produced during the ketogenesis process) promotes protein synthesis [18, 41, 42].

We also found that patients were compliant with the diet protocol based on consistent weight loss and presence of ketonuria in accordance with other studies that attended weight loss with LCKD [43–46].

Traditionally considered high protein, ketogenic diets are often looked at with concern by clinicians due to the potential harm they pose to renal function. Herein, as reported in a recent meta-analysis of Castellana et al [47], we found that LCKD appears safe, considering that not only is it associated to an important improvement in patient’s clinical status but also does not affect renal function.

In the present study, body composition was measured by BIA. We are aware that BIA in severely obese patients has been criticized because of the altered electrical properties in body tissues, which may result in an overestimation of FFM and an underestimation of FM [27, 28]. However, several studies conducted in patients with obesity validate the use of BIA for the measure of body composition [25, 48–53]. Achamrah et al., in a retrospective study on 3655 subjects (653 males, 3002 females) with a body mass index (BMI) ranging from 16 to ≥ 40, found that values of FM and FFM obtained by BIA and DXA were strongly correlated (Pearson’s correlation, *r* = 0.95, *p* < 0.0001, and *r* = 0.89, *p* < 0.0001, respectively) [48]. Furthermore, Faria et al. in a cross-sectional validation study with 73 patients invited to undergo a multi-frequency BIA and afterwards a DXA examination found an almost perfect correlation of FM and FFM (ICC = 0.832 and ICC = 0.899, respectively) [51].

We acknowledge some methodological limitations of our study. First, despite after discharge the physical activity was encouraged, we were not able to directly measure it. Furthermore, FM and FFM were only measured by BIA and were not supplemented with additional and more accurate comparative measures, such as X-ray absorptiometry (DXA) or air displacement plethysmography (ADP) computed tomography (CT). However, Gomez-Arbelaez et al. recently assessed the LCKD-induced changes in body composition of patients with obesity by comparing DXA, BIA, and ADP to evaluate those changes [19]. In this study, similarly to the present research, twenty obese patients followed a VLCK diet for 4 months. After 4 months, the VLCK diet induced a − 20.2 ± 4.5-kg weight loss, at expenses of reductions in fat mass (FM) of − 16.5 ± 5.1 kg (DXA), − 18.2 ± 5.8 kg (MF-BIA), and − 17.7 ± 9.9 kg (ADP). They conclude that a strong correlation was evidenced between the 3 methods of assessing body composition, and that of the 3 body composition techniques used, the MF-BIA method seems to be more convenient in the clinical setting [19].

Nevertheless, DXA requires specialized radiology equipment and is expensive, and thus hardly feasible in routine clinical practice, whereas CT scan is not cost-effective and radiation exposure would not be acceptable for ethical issues. Therefore, despite the fact that we are aware that BIA, DXA, and CT scan methods cannot be considered interchangeable, if the systematic error associated to the measurements of BIA is accepted, the latter remains a simple, safe, non-invasive, and low-cost method for FM and FFM assessment in clinical practice and research studies also in the setting of obesity [25, 48–53].

## Conclusion

This prospective, randomized, controlled trial shows, despite the small sample size, that in patients undergoing EIGB, LCKD is associated with an increased FM loss while reducing the FFM loss and the RMR, without interfering with renal function when compared with LCD. Further and larger randomized clinical trials are needed to confirm these preliminary data.
